# Social and Structural Determinants of Health Inequities: Socioeconomic, Transportation-Related, and Provincial-Level Indicators of Cost-Related Forgone Hospital Care in China

**DOI:** 10.3390/ijerph18116113

**Published:** 2021-06-06

**Authors:** Samuel D. Towne, Xiaojun Liu, Rui Li, Matthew Lee Smith, Jay E. Maddock, Anran Tan, Samah Hayek, Shira Zelber-Sagi, Xiaoqing Jiang, Haotian Ruan, Zhaokang Yuan

**Affiliations:** 1 School of Global Health Management and Informatics, University of Central Florida, Orlando, FL 32816, USA; 2 Disability, Aging, and Technology Cluster, University of Central Florida, Orlando, FL 32816, USA; 3 Department of Environmental and Occupational Health, School of Public Health, Texas A&M University, College Station, TX 77843, USA; matthew.smith@tamu.edu (M.L.S.); maddock@tamu.edu (J.E.M.); 4 Southwest Rural Health Research Center, Texas A&M University, College Station, TX 77843, USA; 5 Center for Population Health and Aging, Texas A&M University, College Station, TX 77843, USA; 6 School of Health Sciences, Wuhan University, Wuhan 430071, China; xiaojun_liu@fjmu.edu.cn (X.L.); rli@whu.edu.cn (R.L.); chloetar@hotmail.com (A.T.); novamadeus@icloud.com (H.R.); 7 Department of Health Management, School of Public Health, Fujian Medical University, Fuzhou 350122, China; 8 Clalit Research Institute, Clalit Health Services, Tuval 40, Ramat-Gan 5252247, Israel; ssamah_shaiek@yahoo.com; 9 School of Public Health, University of Haifa, Haifa 3498838, Israel; shira.zelber@gmail.com; 10 Department of Medical Affairs, Sun Yat-Sen Memorial Hospital of Sun Yat-Sen University, Guangzhou 510275, China; jiangxq6@mail.sysu.edu.cn; 11 School of Public Health, Nanchang University, Nanchang 330031, China; 13576935811@126.com

**Keywords:** health inequities, access, social determinants, costs, hospitals

## Abstract

Despite near universal health insurance coverage in China, populations with low incomes may still face barriers in access and utilization of affordable health care. We aimed to identify the likelihood of forgone medical care due to cost by surveying individuals from the community to assess: (1) The percent with forgone medical care due to cost; and (2) Factors associated with forgone medical care due to cost. Surveys conducted (2016–2017) in Mandarin included demographic and medical care utilization-related items. Theoretically-informed, fully-adjusted analyses were employed. Approximately 94% of respondents had health insurance, which is somewhat similar to national estimates. Overall, 24% of respondents resided in rural areas, with 18% having less than a high school education, and 49% being male. More than 36% reported forgone medical care due to cost in the past 12 months. In fully-adjusted analyses, having lower education, generally not being satisfied with the commute to the hospital, and being a resident of a province with a lower density of physicians were associated with forgone medical care. Cost-related disparities in the access and utilization of needed medical care persist, even with near universal health insurance, which may be due to one’s satisfaction with travel time to healthcare and other community assets.

## 1. Introduction

Forgoing essential medical care due to cost is a major decision faced by many individuals throughout the world [1], and especially burdensome for those living in low-to-middle income countries where the proportion of out-of-pocket spending can be high [2]. A recent World Health Organization (WHO) report noted that nearly 100 million people globally are forced into “extreme poverty” due to health care costs [3]. While access to health care insurance can be critical, the affordability of care and alignment of financing mechanisms within the health care system can affect access to care, even in countries where health insurance is near universal.

Residents of the world’s most populous country, China [4], have seen major reforms in the health care system in recent history, with policies aligned in large part with the goal of increasing access to health care [5]. Moreover, recent estimates indicate that close to 95% of the population in China have some form of health insurance coverage [6]. While there has been much progress, especially since the implementation of major health care system reforms in 2009, there still exist significant opportunities for improvement in terms of increasing access to and utilization of appropriate health care settings and related health care delivery resources [5]. A study on early reforms in China found differential effects from health care reforms with out-of-pocket expenses decreasing largely for those healthier individuals classified as having high income [7]. Another study using more recent data focused on participants of the New Cooperative Medical Scheme (NCMS) identified potential increased outpatient costs among rural residents with the implementation of NCMS outpatient initiative with limited change to NCMS participants’ out-of-pocket payments [8]. Thereby, despite the intended effects of complex reforms, health care reforms may have mixed impacts, depending on the population. Furthermore, previous research using earlier data from China identified an increase in the percent who reported “financial hardship” as the reason for forgone medical care from 1993 to 2003 [9]. Thus, there is a need to explore the potential for- and factors associated with forgone medical care.

### 1.1. Potential Drivers of Forgone Medical Care: Out-of-Pocket Spending Patterns

While a full review of these health care reforms and the broader health care system is outside the scope of this study, several relevant resources provide an overview of China’s health care system [10], and discussion about recent and relevant health-related reforms in China [5,11]. Opportunities for improvement exist even in light of major progress in terms of health insurance coverage (i.e., covering the vast majority of citizens [6]) and reductions in the percent of out-of-pocket expenditures relative to national health expenditures from upwards of 60 to 35% from 2001 to 2011 [6], respectively. For example, more recent data published by the World Health Organization (WHO) Global Health Expenditure Database indicated that the percentage of out-of-pocket expenditures out of “current health expenditures” dropped from 60% in 2000 to 36% in 2016 [12]. Thus, the burden of out-of-pocket cost relative to current health expenditures has made dramatic progress, yet remains relatively high, especially when compared with out-of-pocket expenditures for other country income-rankings, namely in high income countries in which a lower relative percentage of out-of-pocket expenditures (out of current health expenditures) exist (e.g., United Kingdom at 15% in 2016; United States at 11% in 2016) [3]. In addition, globally, health care spending increased between 2000 and 2016 at a rate of approximately 4% for high income countries, with low and middle income countries facing growth of approximately 6% [3]. Thus, with high out-of-pocket costs and increasing health care spending globally, there exists a critical need to identify the likelihood of forgoing essential medical care due to cost and factors that may be associated with such decisions to forgo essential medical care.

### 1.2. Inefficient Utilization Patterns: Utilization of Hospitals for Routine Medical Care

In China, many seek regular health care in hospitals, rather than smaller primary care clinics for routine health care. For example, health care that could otherwise be treated in outpatient settings is routinely received in inpatient settings within hospitals [13]. This is due to a variety of reasons, including but not limited to certain insurance schemes covering inpatient services over outpatient services [13]. Thus, alignment of financing mechanisms within the health care system can affect utilization patterns including the decision to seek health care in settings that may not be aligned with the necessary level of care, potentially leading to overutilization of unnecessary levels of care. In fact, one recent study investigating awareness of the role of general practitioners in China found that just over half of respondents had *not* heard of general practitioners [14] which makes hospitals a critical setting in examinations of health care utilization in China. Thus, when assessing health care utilization patterns in China, it can be particularly relevant to identify health care sought at hospitals.

### 1.3. Transportation and Geospatial Accessibility of Health Care Services

Geospatial measures of access to health care have been shown to play a key role in accessibility. For example, access to transportation, measured in multiple ways, has been tied to increased utilization of health care services in certain rural areas [15]. Furthermore, the geospatial distribution of health care facilities, namely hospitals, has been linked to accessibility to health care in China in prior research [16]. Past research has also suggested that not only increased health care resources, but also improvements with public transportation to hospitals, were needed to increase accessibility to health care in China [17]. Thus, given that transportation has been named a social determinant of health in past research [18], investigations into access to care may benefit from including some measurement of transportation or travel to health care in the analyses of access to care.

### 1.4. Aims

It is clear that the share of health care expenditures that stem from out-of-pocket expenses may continue to impact perceptions on the affordability of health care for many individuals in China. As such, this may impact the individuals’ decision of whether to seek medical care, given the potential cost burden. Given, the role of hospitals in residents seeking even routine health care in China, hospitals serve as an important setting in seeking to identify deficiencies (forgone medical care due to cost) in health care seeking. Thus, we aimed to identify potential variations in the likelihood of reporting forgone medical care due to cost in these settings. We hypothesized that though nearly 5% are without health insurance, given the near universal health care coverage, that there would be higher rates of forgone medical care due to costs. Furthermore, we expected that there would be individual-level and structural or contextual factors related to forgoing medical care due to cost, given the theoretical considerations linking individual-level and structural determinants driving health inequities reported by the World Health Organization (WHO) [19].

## 2. Materials and Methods

### 2.1. Study Setting and Data

Data were collected over the course of approximately 1 year (2016–2017) by members of select author-affiliated institutions. Surveys were carried out in strategic public spaces to capture a diverse participant pool (e.g., parks, train stations, in public buildings) using paper surveys by trained data collectors. Data collectors received instruction from senior team members about how to properly carry out data collection. All participants voluntarily consented (verbally) to participate. For participants with limited reading and/or writing capabilities data collectors read survey items to participants.

The structure of the survey included commuting-related items (e.g., estimated distance to medical facilities), satisfaction with one’s commute to key destinations (e.g., medical facilities), health-related items, and sociodemographics. Respondents reported residing in 20 provinces, most in Jiangxi followed by Hubei.

### 2.2. Dependent Variable

The dependent variable in the current study included a single survey item assessing forgone medical care informed by similar items used in large national surveys [20]. A similar survey item has been used in multiple large-scale surveys [21,22] and noted in the PhenX Toolkit [23]. Participants were asked (translated into Mandarin, with the original item in English as follows): *During the past 12 months, was there any time when you needed any of the following, but didn’t get it because you could not afford it*...This stem was followed by response items including *To go to the hospital* with *Yes* or *No* listed as the answer options.

### 2.3. Individual-Level Variables

In summary, age, sex, education, insurance status, whether one had a primary care physician (PCP) or not, whether the participant resided in an urban or rural area, satisfaction in accessing medical care (their commute to the hospital), and one’s self-estimated travel time (in minutes) to the closest hospital one would use were included as individual-level variables.

Age (continuous) and sex (male or female) were asked. Education was asked as: (1) Less than high school; (2) Some high school, but no degree/diploma/vocational degree; (3) High school diploma/vocational degree; (4) Some college; (5) College degree. This was collapsed into: (1) Less than high school; (2) Some high school, but no degree/diploma/vocational degree; (3) High school diploma/vocational degree; and (4) Some college or above (College degree).

### 2.4. Satisfaction in Accessing Medical Care

In addition, given the study focus on access to medical care, we included a variable that assessed one’s satisfaction with their commute to the hospital. Respondents were asked, *How satisfied are you with: Your commuting time to your hospital*, with responses from *strongly dissatisfied* to *strongly satisfied*. Responses were collapsed into satisfied versus not. Given our theoretical framework tied to structural and social determinants of health inequities [19] and that transportation has been named a social determinant of health in past research [18], this variable was included in the analyses.

While not included in the fully adjusted analyses, we also carried out the analyses cross-referencing a separate variable, one’s self-estimated travel time (in minutes) to the closest hospital one would use, and this above-mentioned variable assessing satisfaction in accessing medical care. This self-estimated travel time (in minutes) variable was limited to responses reporting some number at least 1 min and above. This variable was excluded from the fully adjusted analyses due to the level of missing values (~17%), the fact that it was not statistically significant in the analyses with the major dependent variable, and that its purpose for the current study was restricted to helping better characterize the variable assessing satisfaction in accessing medical care.

### 2.5. Geospatial and Structural Variables

Rurality, whether the participant resided in an urban or rural area, was collected and used as a proxy for resource availability. The province was used to link to provincial-level data. Provincial-level data (2017) were from the National Bureau of Statistics of China (NBSC). These variables included: The number of licensed doctors per 10,000 persons; average wage of employed persons in urban units (yuan); natural population growth rate defined according to the NBSC website as “natural population growth rate = (annual birth population − annual death population)/annual average population × 1000‰ = birth rate-mortality rate” direct source: http://data.stats.gov.cn (accessed on 5 June 2020). The number of licensed doctors per 10,000 persons was used as a rough proxy for access to medical doctors.

### 2.6. Statistical Analyses

SAS version 9.4 (SAS Institute Inc., Cary, NC, USA) was used for all the analyses. Descriptive statistics present the distribution of the data. Logistic regression was employed to assess the binary outcomes across multiple variables. Adjusted logistic regression accounted for multiple variables simultaneously. Generalized linear mixed models (GLMM) were tested, given the clustered nature of the data. The GLMM without predictors (empty model) was used to calculate the intraclass correlation coefficient (ICC). Given similar findings for individual-level variables in multi-level analyses as those from single-level logistic regression and the fact that single-level (logistic regression) models included province-level factors, we highlight results from the simpler, single-level model. The adjusted analyses were informed with three criteria: (1) Statistical significance (*p* < 0.05); (2) The theoretical framework; and (3) The distribution and sample size of our data.

*Theoretical framework.* The theoretical framework used in these analyses was the WHO Framework for Action on the Social Determinants of Health [19]. This framework stipulates that while individual-level characteristics are critically important in predicting potential health inequities, structural determinants (e.g., place-based) also play a critical role. Thus, including both individual-level (e.g., education) and placed-based factors (e.g., satisfaction with transportation to hospitals, rurality, province indicators of the number of licensed doctors per 10,000 residents) was critical to this approach.

*Distribution and sample size.* Additionally, the distribution of the data informed variables to include in the adjusted analyses, given some response items had insufficient cell sizes in the data. For example, the percentage of participants reporting not having a primary care physician (PCP) was less than 7% (n = 50) and also not statistically significant (no difference between those having a PCP versus not for the outcome) in unadjusted analyses. Furthermore, while the insurance status was highly predictive of forgone care, where the lack of insurance was associated (*p* < 0.05) with a higher likelihood of forgone care, the distribution of the variable, with less than 7% (n = 47) of the total sample reporting not having insurance, led to the decision to drop this variable from the adjusted models. The percentage of individuals with health insurance was similar to national estimates [6] and thereby a strength in terms of the generalizability of health insurance coverage in the study sample relative to the distribution in the larger population of China.

## 3. Results

### 3.1. Description of Study Population

Table 1 summarizes the distribution of the data by key characteristics. Of the 760 individuals (see Table 1) that responded to the question about forgone hospital medical care due to cost, the mean age was 35 (range 18–82). Overall, most respondents reported being insured (94%), not having a primary care physician (93%), residing in an urban area (76%), being female (51%), having some college or higher (67%), and being satisfied with their commute to the hospital (53%). When exploring the distribution of one’s self-estimated travel time (in minutes) to the closest hospital one would use and being satisfied or not with their commute to the hospital we found that the estimated travel time (mean = 20.2 min with a median = 15 min, overall) was significantly different (*p* < 0.0001) across levels of satisfaction and highest among those rating their level of satisfaction with their commute as dissatisfied (mean = 30.6 min), followed by neither dissatisfied nor satisfied (mean = 22.2 min) and being satisfied (mean = 15.4 min).

Provincial-level data suggested that most respondents resided in areas with lower relative wages (92%), with a higher relative growth rate (80%), and a lower relative rate of licensed doctors per 10,000 residents as of 2017 (76%).

Overall, 486 respondents did not report forgone medical care due to cost (64%). Of those that were insured, 35% reported forgone medical care in the past 12 months. Of those that did not have a primary care physician, 36% reported forgone medical care in the past 12 months. Among rural residents, 36% reported forgone medical care, which was similar to that among residents of urban areas. Among those residing in a province with a lower relative rate of licensed doctors per 10,000 residents as of 2017, 43% reported forgone medical care in the past 12 months.

### 3.2. Unadjusted Analyses

Table 2 summarizes the unadjusted analyses.

Unadjusted models (Table 2): In terms of the unadjusted likelihood of reporting forgone medical care due to cost, those without health insurance (logistic regression: OR = 2.6, 95% CI 1.4–4.6; multi-level logistic regression: OR = 2.6, 95% CI 1.4–4.9), those with lower education (versus some college or higher) (logistic regression: Less than high school (HS) OR = 3.3, 95% CI 2.3–4.9; some HS OR = 2.3, 95% CI 1.2–4.6; HS diploma/equivalent OR = 2.4, 95% CI 1.5–3.9; multi-level logistic regression: Less than high school (HS) OR = 2.6, 95% CI 1.7–3.8; some HS OR = 2.2, 95% CI 1.1–4.5; HS diploma/equivalent OR = 2.3, 95% CI 1.4–3.8), and those dissatisfied with their commute to the hospital (versus satisfied) (logistic regression: OR = 1.8, 95% CI 1.2–2.7; multi-level logistic regression: OR = 1.7, 95% CI 1.1–2.5) were more likely to report forgone medical care due to cost.

In terms of provincial-level factors, those residing in relatively lower wage areas (OR = 3.5, 95% CI 1.7–7.1), those residing in relatively higher growth areas (OR = 5.3, 95% CI 3.1–8.8), those residing in relatively lower physician density (OR = 4.9, 95% CI 3.1–7.8) were more likely to report forgone medical care due to cost.

### 3.3. Adjusted Analyses

Table 3 summarizes the adjusted analyses.

Adjusted models (Table 3): In terms of the adjusted likelihood of reporting forgone medical care due to cost, those with lower education (versus some college or higher) (logistic regression: Less than high school (HS) OR = 2.7, 95% CI 1.8–4.0; some HS OR = 2.1, 95% CI 1.02–4.2; HS diploma/equivalent OR = 1.9, 95% CI 1.2–3.2; multi-level logistic regression: Less than high school (HS) OR = 2.6, 95% CI 1.7–3.9; some HS OR = 2.1, 95% CI 1.03–4.4; HS diploma/equivalent OR = 2.1, 95% CI 1.3–3.6), and those dissatisfied with their commute to the hospital (versus satisfied) (logistic regression: OR = 1.6, 95% CI 1.04–2.5; multi-level logistic regression: OR = 1.6, 95% CI 1.04–2.5) were more likely to report forgone medical care due to cost.

In terms of provincial-level factors, those residing in relatively lower physician density (OR = 4.0, 95% CI 2.5–6.4) were more likely to report forgone medical care due to cost.

## 4. Discussion

The current study identified significant opportunities for improvement in terms of the existence of forgone medical care due to cost and in terms of identifying factors important for ameliorating inequities in forgone care, even among a largely insured population. Sociodemographic factors such as lower education and perceptions/satisfaction with accessibility of medical care, namely being dissatisfied with ones’ commute to the hospital played a role in the likelihood of reporting forgone medical care due to cost. Furthermore, physician density also played a role in the likelihood of forgone medical care due to cost, where lower relative physician density was associated with a higher likelihood of forgone medical care. Given transportation’s role as a social determinant of health [18], our finding related to satisfaction with transportation to hospitals and more broadly accessibility in terms of available providers further supports the critical role of transportation-related indicators in access to care. Further studies should consider other measures of transportation and related indicators in future analyses of health inequities.

Resources, such as health care providers and transportation, have been characterized as community health care assets [24], and as such play a key role in the overall picture of accessibility to health care. The current findings reinforce the role that both individual-level and structural determinants play in identifying health inequities [19]. Furthermore, while transportation can be measured in terms of distance and/or travel time, there are other considerations such as the cost of one’s time in accessing health care services that may play a role in one’s reported satisfaction. For example, past research has identified that among those that were characterized as having disabilities (e.g., physical), that a majority reported their social life was negatively impacted by transportation-related needs [25]. Other work examining health, transport, and social exclusion identified transportation as being strongly tied to social exclusion [26]. Additionally, the concept of transport disadvantage has also been suggested as a critical target for key stakeholders within public policy [27]. As such, future studies should consider the critical role that not only individual characteristics play, but also the critical role of context or structural characteristics, especially transportation-related indicators, given the multi-faceted role they may play.

Furthermore, less than 7% of the sample reported having a primary care physician, which is an incredible opportunity for improvement. This is not surprising, as the opportunity to improve utilization of and the quality of primary health care in China, has attracted attention by policy makers. For example, overuse of hospitals for minor conditions, stemming from, at least in part, limited coverage for outpatient care or primary care [11] is a major issue and of relevance to the findings in the current study. This also helps reinforce the rationale to focus the current study on forgone hospital care, where much of the care is sought. However, moving towards a more efficient use of resources can include utilization of primary care settings when appropriate and as such should be explored in future work.

Recent existing research specifically assessing forgone medical care in the past 12 months due to cost in the context of both individual-level and structural determinants of health inequities among a broad range of ages in China is rare to the best of our knowledge. One recent study assessing factors associated with forgone care, among a nationally representative sample of middle-aged and older adults in China identified several important factors in predicting forgone medical care (e.g., employment, age, education, marital status) [28]. Though that study was more narrowly focused on middle-aged and older adults [28], similar findings such as education playing a role in forgone care was consistent with the current study. Furthermore, a somewhat recent study identified differential out-of-pocket spending, in terms of the percent of one’s income spent on health care, across income classifications and rural and urban residence in China [9]. That study reported individuals with lower relative incomes in urban and rural areas seeing a higher percent of their income spent on health care relative to their counterparts with higher incomes and that the relative percent was higher in rural areas than in urban areas [9]. While rurality did not appear to play a role in forgone medical care due to cost in the current analyses, future research should continue to investigate this factor, as it is likely to serve as a proxy for not only access to care, but also socioeconomic status.

### Limitations and Strengths

Identifying trends over time was not the goal of the current study and as such we used a cross-sectional approach. This does not allow for the assessment of trends over time or identifying causality, thereby limiting the study in that regard. Furthermore, the sampling approach was not the type that allows for the data to be nationally representative, another limitation. As with any similar study design, the implications of the current study may not be generalizable to the larger Chinese population. That said, there were several strengths in terms of the distribution of the data. For example, respondents reported residing in 20 provinces throughout China providing some geospatial spread. In addition, insurance status, which is relevant to our study outcome, did have a somewhat similar distribution in our sample as that of the larger Chinese population [6]. In addition, the mean (35 years) and median age (31 years) in our study was somewhat similar to the median age of China in 2015 at approximately 37 years [4]. Additionally, the percent female at 51% in our study was also roughly similar to that of China overall, at approximately 49% [29]. Furthermore, we did include several relevant factors related to our outcome, as informed by our theoretical framework, which, coupled with the relatively large sample size allowed for analyses including subgroups and several key variables.

The intensity of forgone medical care due to cost was not measured, given the question, as included in country-wide surveys carried out elsewhere [20], only asked if there was at least one time that the individual did not seek care due to cost. Thus, we could not differentiate the number of times this occurred, which may serve as a limitation. That said, several strengths exist, as well. For example, while the number of times one experienced forgone medical care due to cost was not measured, the question does allow for analyses of a specific (i.e., due to cost) and critical reason for forgone medical care. Furthermore, this study sought to identify forgone medical care due to cost in the context of care that might be sought at hospitals. As such, forgone medical care due to cost that might be sought at other types of facilities was not included. Furthermore, comparisons to the existing literature were largely restricted to English-language publications, though estimates indicate much of the existing literature is available in English-language journals [30]. These and other limitations should be considered in light of the implications.

## 5. Conclusions

The current study identified that sociodemographic factors, perceptions/satisfaction with accessibility of medical care, and structural factors play a critical role in forgone medical care due to cost for a population with near universal health insurance. While health insurance is likely critical in the individuals’ decisions to seek out affordable health care, it is clearly not only the presence of *some* form of health insurance, but also several other relevant components such as out-of-pocket costs. In the adjusted analyses, having lower education, generally not being satisfied with the commute to the hospital, and being a resident of a province with a lower density of physicians were associated with forgone medical care. Thus, forgoing medical care, even in the presence of widespread health insurance, in some form, can be both multifaceted and in need of complex solutions. This study and the implications found herein framed in light of the limitations, can serve to inform future research that can further investigate and identify relevant factors related to forgone medical care due to cost. The existence of such knowledge, in combination with past and future studies, can inform targeted policy interventions with the aim of decreasing the likelihood of inequities in forgone medical care.

## Figures and Tables

**Table 1:** Description of the study population overall and by forgone medical care.

		Overall		Forgone Medical Care			
				No		Yes	
		**n**	**Mean (Median)**	**n**	**Mean (Median)**	**n**	**Mean (Median)**
**Age**		760	34.97 (31.00) years	486	32.02 (26.00) years	274	40.19 (42.00) years
		**n**	**Percent**	**n**	**Percent**	**n**	**Percent**
Insurance Status	Insured	705	93.75	461	61.30	244	32.45
	Not Insured	47	6.25	20	2.66	27	3.59
Primary Care Physician (PCP)	Not having a PCP	702	93.35	450	59.84	252	33.51
	Having a PCP	50	6.65	32	4.26	18	2.39
Rurality	Rural	185	24.34	118	15.53	67	8.82
	Urban	575	75.66	368	48.42	207	27.24
Sex	Female	389	51.18	252	33.16	137	18.03
	Male	371	48.82	234	30.79	137	18.03
Education	Less than High School	137	18.03	60	7.89	77	10.13
	Some High School	36	4.74	19	2.50	17	2.24
	High School Degree or equivalent	81	10.66	42	5.53	39	5.13
	Some College or Higher	506	66.58	365	48.03	141	18.55
Satisfaction with Commute to Hospital	Not Satisfied	127	16.71	66	8.68	61	8.03
	Neither Dissatisfied nor Satisfied	227	29.87	151	19.87	76	10.00
	Satisfied	406	53.42	269	35.39	137	18.03
Provincial Wage (2017)	Low	700	92.11	435	57.24	265	34.87
	High	60	7.89	51	6.71	9	1.18
Growth Rate (2017)	Low	149	19.61	131	17.24	18	2.37
	High	611	80.39	355	46.71	256	33.68
Licensed Doctors per 10,000 residents (2017)	Low	580	76.32	330	43.42	250	32.89
	High	180	23.68	156	20.53	24	3.16

Note: Response distribution for forgone medical care: No, with n = 486; Yes, with n = 274, where percentages are calculated excluding missing data which may vary per variable.

**Table 2:** Unadjusted analysis for the likelihood of forgone care.

		Logistic Regression			Generalized Linear Mixed Models		
		OR	95% Confidence Intervals		OR	95% Confidence Intervals	
Insurance Status	Not Insured versus Insured	2.550 *	1.401	4.640	2.572 *	1.353	4.888
Primary Care Physician (PCP)	Not having a PCP versus having a PCP	0.996	0.548	1.810	1.049	0.562	1.960
Rurality	Rural versus Urban	1.009	0.715	1.425	0.981	0.682	1.410
Sex	Female versus Male	0.929	0.691	1.249	0.798	0.583	1.092
Education	Less than High School versus Some College or Higher	3.321 *	2.250	4.903	2.558 *	1.709	3.829
	Some High School or equivalent versus Some College or Higher	2.316 *	1.170	4.583	2.182 *	1.065	4.470
	High School diploma or equivalent versus Some College or Higher	2.403 *	1.491	3.873	2.285 *	1.379	3.787
Satisfaction with Commute to Hospital	Dissatisfied versus Satisfied	1.815 *	1.211	2.719	1.645 *	1.073	2.521
	Neither Satisfied nor Dissatisfied versus Satisfied	0.988	0.701	1.394	0.913	0.637	1.309
Provincial-level variables							
Provincial Wage (2017)	Low versus High	3.452 *	1.672	7.126	-	-	-
Growth Rate (2017)	High versus Low	5.248 *	3.126	8.812	-	-	-
Licensed Doctors per 10,000 residents (2017)	At/Lower than the Upper Quartile versus Higher	4.923 *	3.107	7.798	-	-	-

* Significantly different (*p* < 0.05). Note: Generalized linear mixed model (GLMM) intraclass correlation coefficient (ICC) = 0.2262; calculated from the empty model. The GLMM model accounts for province-level variation and as such, province-level variables are not presented.

**Table 3:** Adjusted analysis for the likelihood of forgone care.

		Logistic Regression			Generalized Linear Mixed Model		
		OR	95% Confidence Intervals		OR	95% Confidence Intervals	
Rurality	Rural versus Urban	0.861	0.588	1.261	0.837	0.568	1.233
Sex	Female versus Male	0.872	0.635	1.197	0.837	0.606	1.155
Education	Less than High School versus Some College or Higher	2.665 *	1.767	4.020	2.604 *	1.720	3.943
	Some High School or equivalent versus Some College or Higher	2.082 *	1.022	4.239	2.125 *	1.029	4.386
	High School diploma or equivalent versus Some College or Higher	1.941 *	1.182	3.187	2.141 *	1.283	3.572
Satisfaction with Commute to Hospital	Dissatisfied versus Satisfied	1.598 *	1.038	2.459	1.613 *	1.039	2.504
	Neither Satisfied nor Dissatisfied versus Satisfied	0.990	0.687	1.426	0.974	0.673	1.411
Provincial-level variables							
Licensed Doctors per 10,000 residents (2017)	At/Lower than the Upper Quartile versus Higher	3.996 *	2.491	6.410	-	-	-

* Significantly different (*p* < 0.05). The GLMM model accounts for province-level variation and as such, province-level variables are not presented. Note: Fully adjusted analyses was based on complete data for each variable included in the model (n = 760), excluding observations deleted due to missing values for the response or explanatory variables.

## Data Availability

Data are protected by IRB protocols and are not publicly available.
